# Complete Genome of the Starch-Degrading Myxobacteria *Sandaracinus amylolyticus* DSM 53668^T^

**DOI:** 10.1093/gbe/evw151

**Published:** 2016-06-29

**Authors:** Gaurav Sharma, Indu Khatri, Srikrishna Subramanian

**Affiliations:** Protein Science and Engineering, CSIR-Institute of Microbial Technology, Sector-39A, Chandigarh, India

**Keywords:** CAZyme, amylase, agarase, cellulase, methylome, phylogeny

## Abstract

Myxobacteria are members of δ-proteobacteria and are typified by large genomes, well-coordinated social behavior, gliding motility, and starvation-induced fruiting body formation. Here, we report the 10.33 Mb whole genome of a starch-degrading myxobacterium *Sandaracinus amylolyticus* DSM 53668^T^ that encodes 8,962 proteins, 56 tRNA, and two rRNA operons. Phylogenetic analysis, in silico DNA-DNA hybridization and average nucleotide identity reveal its divergence from other myxobacterial species and support its taxonomic characterization into a separate family Sandaracinaceae, within the suborder Sorangiineae. Sequence similarity searches using the Carbohydrate-active enzymes (CAZyme) database help identify the enzyme repertoire of *S. amylolyticus* involved in starch, agar, chitin, and cellulose degradation. We identified 16 α-amylases and two γ-amylases in the *S. amylolyticus* genome that likely play a role in starch degradation. While many of the amylases are seen conserved in other δ-proteobacteria, we notice several novel amylases acquired via horizontal transfer from members belonging to phylum Deinococcus-Thermus, Acidobacteria, and Cyanobacteria. No agar degrading enzyme(s) were identified in the *S. amylolyticus* genome. Interestingly, several putative β-glucosidases and endoglucanases proteins involved in cellulose degradation were identified. However, the absence of cellobiohydrolases/exoglucanases corroborates with the lack of cellulose degradation by this bacteria.

## Introduction

Myxobacteria [order Myxococcales] are Gram-negative and aerobic (except *Anaeromyxobacter*) δ-proteobacteria. These bacteria possess large genomes and a complex life cycle wherein they coordinate within swarms and hunt other microbes (Shimkets 1990;Whitworth 2008). Myxobacterial species have been of industrial interest due to their significant role in secondary metabolites production (Weissman and Muller 2010; Steinmetz et al. 2012). Order Myxococcales at present comprises of more than 27 taxonomically classified genera and 60 species. This order has been classified in three suborders, Cystobacterineae (four families), Nannocystineae (two families), and Sorangiineae (four families). The members of suborder Sorangiineae are cellulolytic, bacteriolytic, and proteolytic as compared with other family members and form a separate clade in myxobacterial 16S rRNA-based phylogeny (Reichenbach 2005; Garcia and Müller 2014). Till date the largest complete bacterial genomes *Sorangium cellulosum* strain So0157-2 (14.78 Mb) (Han et al. 2013) and *S. cellulosum* strain Soce56 (13.03 Mb) (Schneiker et al. 2007) have been reported from the suborder Sorangiineae. These members are known to degrade diverse, complex polysaccharides such as cellulose, starch, casein, agar, chitin, gelatin, dextrin, xylan, agarose, etc. (Garcia and Müller 2014). They have also been reported as microbial predators to organisms such as *Escherichia coli*, *Bacillus subtilis*, *Mycobacterium chitae*, Cyanobacteria, yeast, etc. (Garcia and Müller 2014). Among the four characterized families of the suborder Sorangiineae, genomic data are available only for *Sorangium* and *Chondromyces* that belong to Polyangiaceae family which are the largest source of myxobacterial secondary metabolites (Schneiker et al. 2007;Weissman and Muller 2010;Han et al. 2013).

Within suborder Sorangiineae, *Sandaracinus amylolyticus* strain DSM 53668^T^ is the type strain of *S. amylolyticus* gen*.* nov*.*, sp*.* nov*.*, proposed as a novel species in the family Sandaracinaceae within order Myxococcales (Mohr et al. 2012). Morphologically and biochemically the strain is a Gram-negative, aerobic, rod-shaped during vegetative states, catalase-negative, oxidase-positive, and pigment forming myxobacteria (Mohr et al. 2012). This strain was isolated in 1996 from a gray-brown soil sample (having plant residues) collection by Helmholtz Zentrums für Infektionsforschung [HZI] during 1995 from a botanical garden in Lucknow, Uttar Pradesh, India (Reichenbach and Dworkin 1992; Mohr et al. 2012)*. Sandaracinus amylolyticus* possesses typical myxobacterial characteristics including gliding motility, secondary metabolite production (Steinmetz et al. 2012), and spore generation during harsh environmental conditions. Here, we report the complete genome of *S. amylolyticus* DSM 53668^T^ with special emphasis on the starch degrading properties along with the protein repertoire involved in starch, agarose, chitin, and cellulose degradation.

## Materials and Methods

### Culturing and Genomic DNA Isolation

Actively growing plate cultures of *S. amylolyticus* was procured from Deutsche Sammlung von Mikroorganismen und Zellkulturen (DSMZ) as strain number DSM 53668^T^ [NOSO-4^T^]*. Sandaracinus amylolyticus* DSM 53668^T^ was grown aerobically on CY agar plate culture (DSM Medium: 67, as prescribed by DSMZ culture collection) at 28–30 °C for a minimum of 5 days. The culture was yellow-orange in color attributed to the presence of carotenoids (Mohr et al. 2012). DNA for sequencing was obtained using both the Zymogen Research Bacterial/fungal DNA isolation kit and Phenol–Chloroform–Isoamyl Alcohol-based manual method. The quantity and quality of the extraction were checked by gel electrophoresis along with Nanodrop method and followed by Qubit quantification.

### Genome Sequencing, Assembly, and Annotation

*Sandaracinus amylolyticus* was sequenced on four single molecule real-time (SMRT) cells using P6 polymerase and C4 chemistry (P6C4) on a Pacific Biosciences RSII instrument at McGill University and Genome Quebec Innovation Center, Montréal (Québec), Canada. DNA sample was sheared and concentrated using AMPure magnetic beads and treated by ExoVII to remove single stranded ends. SMRTbell long libraries were constructed with 10 µg whole genomic DNA using “Procedure and Checklist–20 kb Template Preparation Using BluePippin^TM^ Size Selection System protocol” (http://www.pacb.com/wp-content/uploads/2015/09/Shared-Protocol-20-kb-Template-Preparation-Using-BluePippin-Size-Selection-System-15-kb-Size-Cutoff.pdf, last accessed July 5, 2016). Blunt ligation reactions were prepared, and SMRTbell templates were purified using AMPure magnetic beads. BluePippin^TM^ size selection was performed to retain long reads (>20,000) for sequencing. The size-selected SMRTbell templates were annealed and then loaded onto four SMRT cells and sequenced using P6C4 chemistry with 180-min movie times. Sequencing yielded a total of 241,674 reads with a mean read length of 4.67 kb, totaling 1,129,177,817 bp (∼85× coverage). De novo assembly was carried out using the hierarchical genome assembly process (HGAP) protocol in SMRT Analysis v2.0, including consensus polishing with Quiver (Chin et al. 2013). Since the BluePippin^TM^ Size Selection (>20,000) protocol filters out small DNA fragments such as plasmids, Illumina paired-end (PE) data were also obtained using the Illumina HiSeq1000 sequencing platform at the C-CAMP, Bangalore, India. Sequencing resulted in 13,103,763 PE reads (insert size: 350 bp and read length: 101 bp) out of which 11,663,870 PE reads were selected after quality filtering [PHRED quality score: 20; minimum read length: 70] using NGSQC Toolkit v2.2.3 (Patel and Jain 2012). To trace the presence of any plasmid, the filtered Illumina reads were mapped using CLCbio wb8.0 (www.clcbio.com, last accessed July 8, 2016) to the bacterial plasmid database (http://www.ebi.ac.uk/genomes/plasmid.html, last accessed July 8, 2016).

Gene prediction and functional annotation were performed by Rapid Annotation using Subsystem Technology (RAST) (Aziz et al. 2008). RNAmmer 1.2 (Lagesen et al. 2007) and tRNAscan-SE-1.23 (Lowe and Eddy 1997) were used to predict rRNA and tRNA genes, respectively.

### Phylogenetic Analysis, Genome-Genome Distance, and Average Nucleotide Identity

16S rRNA genes from various strains of suborder Sorangiineae along with neighboring families were extracted from the National Centre for Biotechnology Information (NCBI), and were aligned using ClustalW module of BIOEDIT sequence alignment tool (version 7.1.3.0; Hall 1999). The resulting alignment was used as an input in Molecular Evolutionary Genetics Analysis (MEGA) v6.06 (Tamura et al. 2013) to generate a maximum likelihood (ML) tree [model: Tamura 3-param; bootstrap: 100]. Initial tree(s) for the heuristic search were obtained by applying the Neighbor-Joining method to a matrix of pairwise distances estimated using the Maximum Composite Likelihood approach. In silico DNA-DNA hybridization (DDH) values among the members of suborder Sorangiineae and other species members of order Myxococcales were calculated using Genome-To-Genome Distance Calculator (GGDC) server (Auch et al. 2010). Average Nucleotide Identity (ANI) matrix was also generated among the genomes studied (Richter and Rossello-Mora 2009).

### Genome and Proteome Analysis

*Sandaracinus amylolyticus* proteome was subjected to hmmscan (Eddy 2011) against Hidden Markov Model (HMM) profiles of Carbohydrate-Active enZYmes (CAZY) database (dbCAN HMMs 3.0; Yin et al. 2012;Lombard et al. 2014). The functional domains and other known sequence motifs involved in starch, cellulose, chitin, and agar degradation were retrieved on the basis of EC number for each enzyme category. Also, the proteins of *S. amylolyticus* were mapped against the CAZY protein database using Basic-Local Alignment Search Tool (BLASTp) (E-value cutoff of 1e^−5^; minimum query coverage of 50% and minimum percent identity of 35%) (Altschul et al. 1990). NCBI BLAST + (v 2.2.26+) and nonredundant protein (NR) database downloaded on September 6, 2014 was used throughout the study. Clustered regularly interspaced short palindromic repeat (CRISPR) elements and insertion elements were identified using CRISPRfinder (Grissa et al. 2007) and ISfinder (Siguier et al. 2006), respectively.

### *Sandaracinus amylolyticus* Amylases

The α-amylases and γ-amylases from *S. amylolyticus* were subjected to the BLASTp against amylase sequences from the CAZY database (clustered at 70% sequence identity) and top hits were retrieved. The amylases from *S. amylolyticus* were cut into the domains and were aligned using MUltiple Sequence Comparison by Log-Expectation (MUSCLE) (Edgar 2004) incorporated in MEGA v6.06. Further the evolutionary tree was generated by ML method [model: Jones, Taylor, and Thorton; bootstrap: 100] using MEGA v6.06. Initial tree(s) for the heuristic search were obtained by applying the Maximum Parsimony method to a matrix of pairwise distances estimated using the Maximum Composite Likelihood approach. Further, the *S. amylolyticus* GH13 amylases along with the closely related members of each phylogenetic clade were aligned using PROfile Multiple Alignment with predicted Local Structures and 3D constraints (PROMALS3D) with Taka-Amylase A of *Aspergillus oryzae* [2TAA] (Matsuura et al. 1984) as a structural template (Pei et al. 2008).

## Results and Discussion

### *Sandaracinus amylolyticus* Genome Properties

**Fig. 1.—:**
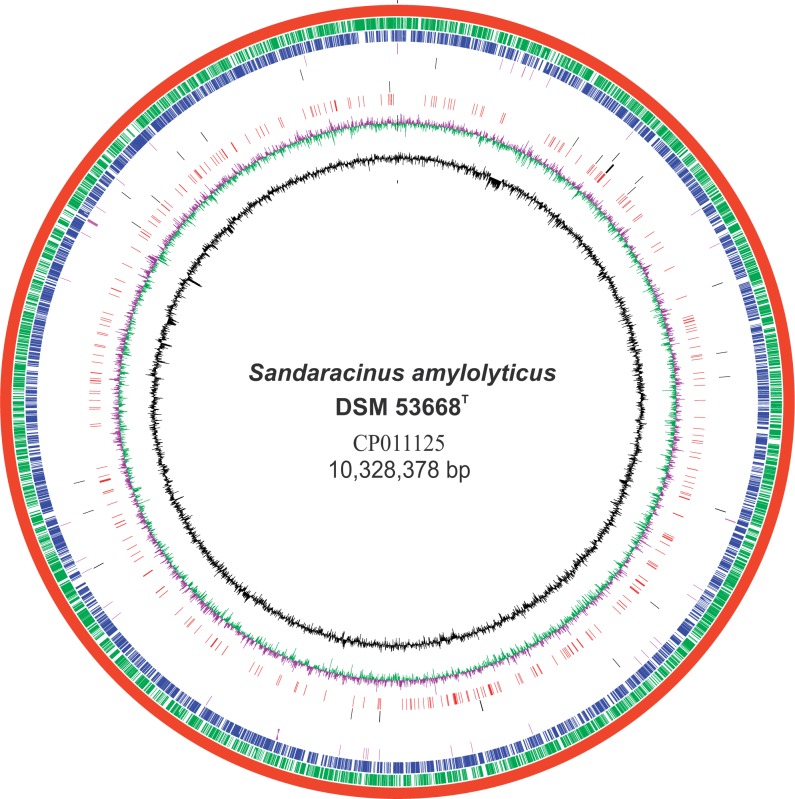
Circular representation of the *Sandaracinus amylolyticus* DSM 53668^T^ complete genome. **Circles** (from inside to outside) **1 and 2** (GC content; black line and GC skew; magenta and green lines), **circle 3** (CAZome: Proteins annotated as carbohydrate active enzymes; Red); **circle 4, 5, and 6** (Amylases, Cellulases, and Chitinases; Black); **circle 7** (encoded RNA, Magenta); **circle 8** (CDS present on negative strand; Blue); **circle 9** (CDS present on positive strand; Green); **circle 10** (*S. amylolyticus* DSM 53668^T^ complete genome; Red). BRIG 0.95 was used to build the circular representation (Alikhan et al. 2011).

*Sandaracinus amylolyticus* genome was assembled de novo by HGAP v2.0 (Chin et al. 2013) of the SMRT portal as a single contig of 10.33 Mb with no plasmid and representing the complete genome with approximately 85× coverage and 72% G+C content (fig. 1). No complete plasmid could be retrieved from the mapping of Illumina PE reads against the bacterial plasmid database. Plasmids are rare in the order Myxococcales and till date only one plasmid pMF1 from *Myxococcus fulvus* 124B02 has been reported (Zhao et al. 2008). RAST-based annotation predicted 8,962 coding genes, out of which 4,939 proteins (55.16%) were functionally annotated while the remaining (44.84%) were hypothetical proteins (supplementary table S1, Supplementary Material online)*. Sandaracinus amylolyticus* genome has two rRNA operons and 56 aminoacyl-tRNA synthetase genes.

This genome encodes two sequentially identical 16S rRNA which shows 100% identity with previously reported 16S rRNA sequence of *S. amylolyticus* strain NOSO-4^T^ [NCBI Reference Sequence: NR_118001.1] followed by 87.98% identity with *Chondromyces apiculatus* strain Cm a14 [NR_025344.1]. Among the myxobacteria with sequenced genomes, it shows the maximum identity (87.89%) to the 16S rRNA gene of *C. apiculatus* DSM 436 (strain Cm a2) [GenBank: M94274.2] followed by 87.5% identity to *S. cellulosum* 16S rRNA (Soce0157-2 and Soce56). The 16S rRNA-based phylogeny of *Sandaracinus* and related myxobacteria suggest its classification as a separate family within the suborder Sorangiineae (supplementary fig. S1, Supplementary Material online)*. Sandaracinus amylolyticu*s 16S rRNA forms a separate branch distinct from other families in the suborder Sorangiineae phylogenetic tree, representing family Sandaracinaceae. We also calculated in silico DDH values for all available myxobacterial genomes using the GGDC server (Auch et al. 2010)*. Sandaracinus amylolyticus* is not closely related to other Myxococcales as the maximum DDH value was identified to be 18.1 with *S. cellulosum* followed by 17.4 with *C. apiculatus* DSM 436 suggesting a distant relation to Polyangiaceae members, which further confirms its placement in a separate family, Sandaracinaceae. ANI calculations likewise support the above conclusions (supplementary table S2, Supplementary Material online).

The members of suborder Sorangiineae such as the *S. cellulosum* species have been well characterized for degrading cellulose and other complex polysaccharides (Schneiker et al. 2007; Han et al. 2013). Similarly, *S. amylolyticus* has been reported as the only myxobacteria capable of degrading starch (Mohr et al. 2012). Thus in order to gain insights into the genes involved in the degradation of complex polysaccharides, especially starch the complete genome of *S. amylolyticus* was determined and investigated for the presence of carbohydrate active enzymes.

### *Sandaracinus amylolyticus* CAZome

**Fig. 2.—:**
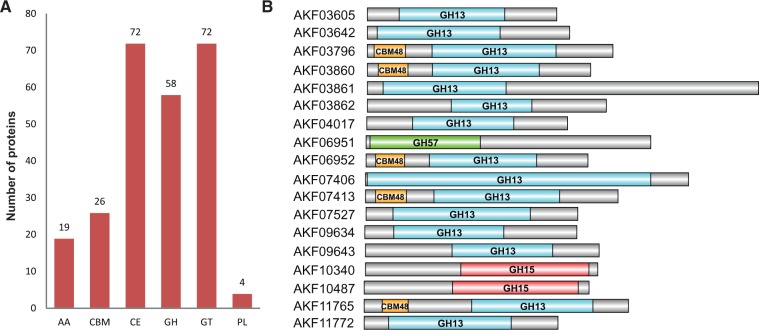
*Sandaracinus amylolyticus* DSM 53668^T^ CAZome distribution. (*A*) *Sandaracinus amylolyticus* DSM 53668^T^ CAZY families presented in a bar graph. *x* axis represents the CAZY categories and *y* axis represents the number of proteins. (*B*) α- and γ-amylases along with their CAZome domain architecture. The domains have been colored as GH13: sky blue; GH15: dark red; GH57: green; and CBM48: orange.

Two-hundred and forty-three proteins (∼2.7% of total proteome) of *S. amylolyticus* were annotated with diverse combinations of CAZY domains that consist of 72 GT domains, 72 CE domains, 58 GH domains, 19 AA domains, 4 PL domains, and 26 CBM domains (fig. 2A). Fourteen out of these 243 proteins contained more than one CAZY domain, whereas the rest had only a single CAZY domain. In multiple CAZY-domain containing proteins, various carbohydrate binding modules such as CBM48, CBM50, and CBM21 are present along with other CAZY category proteins for assisting in their functions. The CAZyme distribution observed in *S. amylolyticus* indicates its ability to degrade various types of biomass. Here, we examine the *S. amylolyticus* proteome involved in starch, agar, chitin, and cellulose degradation.

### Evolutionary History of *S. amylolyticus* Amylases

Amylases, belonging to glycoside hydrolase (GH) enzymatic category, degrade starch into sugar moieties by the hydrolysis of α-1,4- and α-1,6-glycosidic bond (Van der Maarel et al. 2002). Based on the site of action, they are divided into three categories; α-amylase (EC:3.2.1.1; GH13, GH57, GH119, GH126), β-amylase (EC:3.2.1.2; GH14), and λ-amylase (EC:3.2.1.3; GH15, GH97)*. Sandaracinus amylolyticus* genome contains both α- and λ-amylases whereas no β-amylases could be found. Among the 4 α-amylases GH families, 15 GH13 proteins, and 1 GH57 protein were found. We could identify two λ-amylases belonging to GH15 category (fig. 2B). The starch degrading proteins have been reported to have CBM20, CBM21, CBM25, CBM26, CBM34, CBM41, CBM45, CBM48, CBM53, and CBM58 modules that help in glycogen/starch binding, truncation of which reduces its enzymatic activity (Noguchi et al. 2011; Janecek et al. 2014). GH15 and GH57 domains are present as single domain proteins in *S. amylolyticus*. Interestingly, in 5 out of 15 GH13 *S. amylolyticus* proteins (AKF03796, AKF03860, AKF06952, AKF07413, and AKF11765), the α-amylase GH13 domain was present along with a CBM48 module at the N-termini. The CBM48 module assists in the GH13 function by serving as starch-binding domains.

The GH13 family include the α-amylase domains with an (β/α)_8_ barrel catalytic domain. These proteins have 4–7 conserved sequence regions (CSRs) and a specific catalytic triad consisting of a catalytic nucleophile (aspartic acid) on strand β4, proton donor (glutamic acid) on strand β5, and a transition state stabilizer (aspartic acid) on strand β7 (Janecek et al. 2014). GH13 members show extremely low sequence identity with their homologs, but the catalytic triad is highly conserved. GH57 family α-amylases have a (β/α)_7_ barrel structure and have five CSRs as compared with the GH13 family. However, only two of the catalytic residues, *viz.*, the nucleophile on strand β4 and the proton donor on strand β7 are conserved (Blesak and Janecek 2012). Irrespective of these differences, GH13, and GH57 both employ the same retaining mechanism of action (Janecek and Kuchtova 2012)*. Sandaracinus amylolyticus* GH13 proteins were retrieved, and the structure of Taka amylase of *A. oryzae* (PDBid: 2TAA) was used to perform a structure-based alignment using PROMALS3D (Matsuura et al. 1984; Pei et al. 2008). All the seven CSR regions and the three catalytic residues were conserved in all *S. amylolyticus* GH13 amylases (supplementary figs. S2 and S3, Supplementary Material online). Similarly, *S. amylolyticus* GH57 (AKF06951) was aligned with its homologs using PROMALS3D based on the 4-α-glucanotransferase structure of *Thermococcus litoralis* (PDBid: 1K1W). In the GH57 alignment, all five CSRs and both catalytic residues were conserved (supplementary fig.S4, Supplementary Material online).

**Fig. 3.—:**
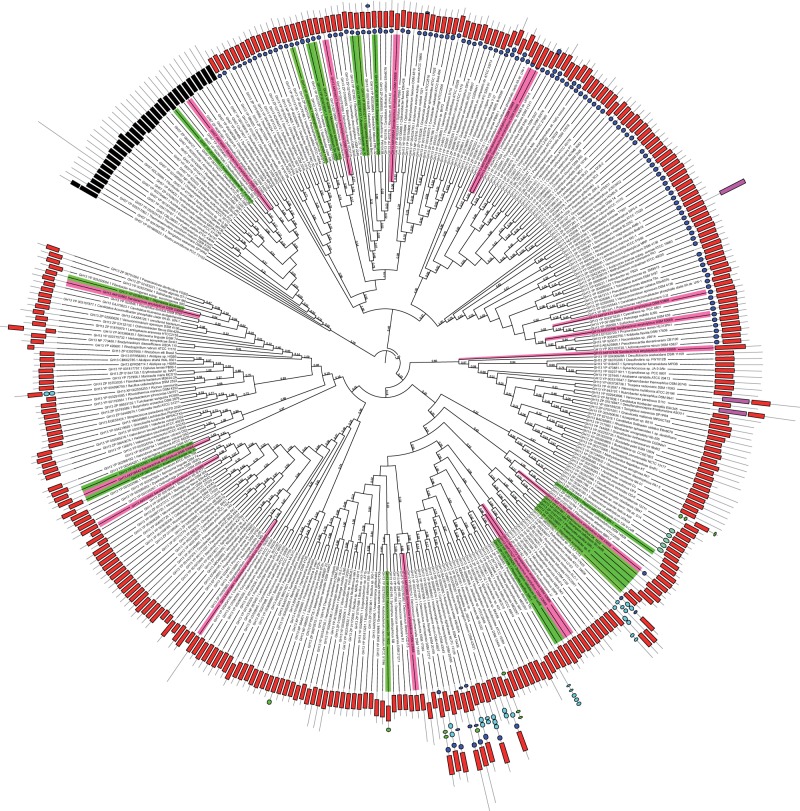
Phylogeny of *Sandaracinus amylolyticus* GH13 and GH57 α-amylases*. Sandaracinus amylolyticus* α-amylases (GH13 and GH57) are colored in pink along with their domain architecture displayed as Domain: Color Shape [GH13: Red, rectangle; GH57: Black, rectangle; GH77: dark pink, rectangle; CBM48: Dark blue, circle; CBM20: green, circle; CBM41: cyan, circle]. Myxobacterial homologs are colored fluorescent green. Bootstrap values corresponding to the tree nodes are provided.

In order to understand the provenance of these α- and γ-amylases, they were subjected to BLASTp against the NR database. To include amylases across the entire bacterial phylum, the CAZY-NR database for GH13 and GH57 domains were retrieved and subjected to BLASTClust at 70% identity and 70% length coverage that reduced the domain sequence space from 19,590 to 4,038 domains. Further, amylase domains from *S. amylolyticus* were subjected to BLASTp against these 4,038 domains, and their top hits were retrieved and included in the phylogenetic analysis. All these GH13 and GH57 domains along with *S. amylolyticus* amylase domains were aligned using MUSCLE as incorporated in MEGAv6.0.6, and ML-based phylogeny with 100 bootstrap values was drawn (fig. 3). The ML tree obtained was divided into three major clades where the clade at root composed of all the GH57 domains and the other two major clades further composed of all the GH13 sequences. The representative bacterial amylases from various clades show close relations with amylases of diverse bacterial groups suggesting the multiple gain and loss of these domains. As all the amylase domains of *S. amylolyticus* are scattered across the tree, the phylogenetic position of these domains could provide us insights about their provenance and reveal possible cases of horizontal transfers.

Taking into account the GH57 clade, the sole GH57 domain of *S. amylolyticus* (GenBank ID: AKF06951) shares the clade with anaerobic Deltaproteobacteria. Among the GH13 domains of *S. amylolyticus*, AKF03862, AKF07527, AKF09643, AKF11765, and AKF11772 shares clade with other myxobacteria. Among these, AKF11772 and AKF07527 share sister branches and form sister clades with other myxobacterial species such as *S. cellulosum* Soce56 and *Plesiocystis pacifica* SIR-1 and are further related to the clade containing Gammaproteobacteria. Apart from these two amylases AKF03860 and AKF06952 are similar to each other and group with *Candidatus accumulibacter phosphtis* amylase (YP_0031672861) from the phylum Acidobacteria. Interestingly, AKF03860 and AKF06952 group with members of the phyla Betaproteobacteria suggestive of a possible horizontal transfer.

AKF04017 and AKF03605 have close homologs in the extremophile *Deinococcus desserti* VCD115 and shares clade with certain myxobacterial amylases belonging to *Myxococcus xanthus*, *S. cellulosum*, and *Haliangium ocraceum* which further share clades with *Deinococcus* amylases. AKF04017 and AKF03605 share the highest sequence identity with *D. desserti* VCD115 plasmid protein (YP_002787899; 53.3%) and chromosomal protein (YP_002785382; 45.6%), respectively, that might point to its plasmid-borne horizontal transfer across the clade (fig. 3). The sequence conservation of CSRs and the high percentage identity of these amylases to *Deinococcus* support the inference for the putative horizontal transfers among these members. AKF09634 does not share its sister branch with any member, but the clade contains members from diverse phyla, that is, *Rhizobium* from alphaproteobacteria, *Opitutus* from Chlamydae, *Chthonibacter* from Verrucomicrobia suggesting the multiple gain and loss of this amylase among prokaryotes. Similarly, AKF07413 shares the clade with members of Cyanobacteria; AKF07406 shares the clade with members of Deltaproteobacteria; AKF03861 with members of Alphaproteobacteria and AKF03642 with members of Bacteroidetes. Taken together, we can suggest that many of the *S. amylolyticus* amylases have been acquired via horizontal gene transfer events from other bacterial species belonging to the phylum Deinococcus-Thermus, Acidobacteria, Cyanobacteria, and Alphaproteobacteria.

To further support these horizontal transfer events, we checked the sequence conservation between *S. amylolyticus* amylases and closely related members in the phylogenetic tree and aligned using PROMALS-3D with Taka-Amylase A of *A. oryzae* (PDBid 2TAA) as a structural template. We also performed synteny studies analyzing proteins present in the vicinity of all identified *S. amylolyticus* GH13 and GH57 amylases and their closely related homologs as revealed by phylogenetic studies. We could not trace any conserved syntenic regions in these genomes whereas in the case of AKF03862 and AKF09634 we identified glycogen debranching enzymes in *Stigmatella aurantiaca* DW4/3-1 [56% identity and 98% query coverage of AKF03860 with WP_013376091.1] and *Opitutus terrae* PB90-1 [57% identity and 97% query coverage of AKF09635 YP_001820542.1] which further support their putative horizontal transfer in *S. amylolyticus*.

**Fig. 4.—:**
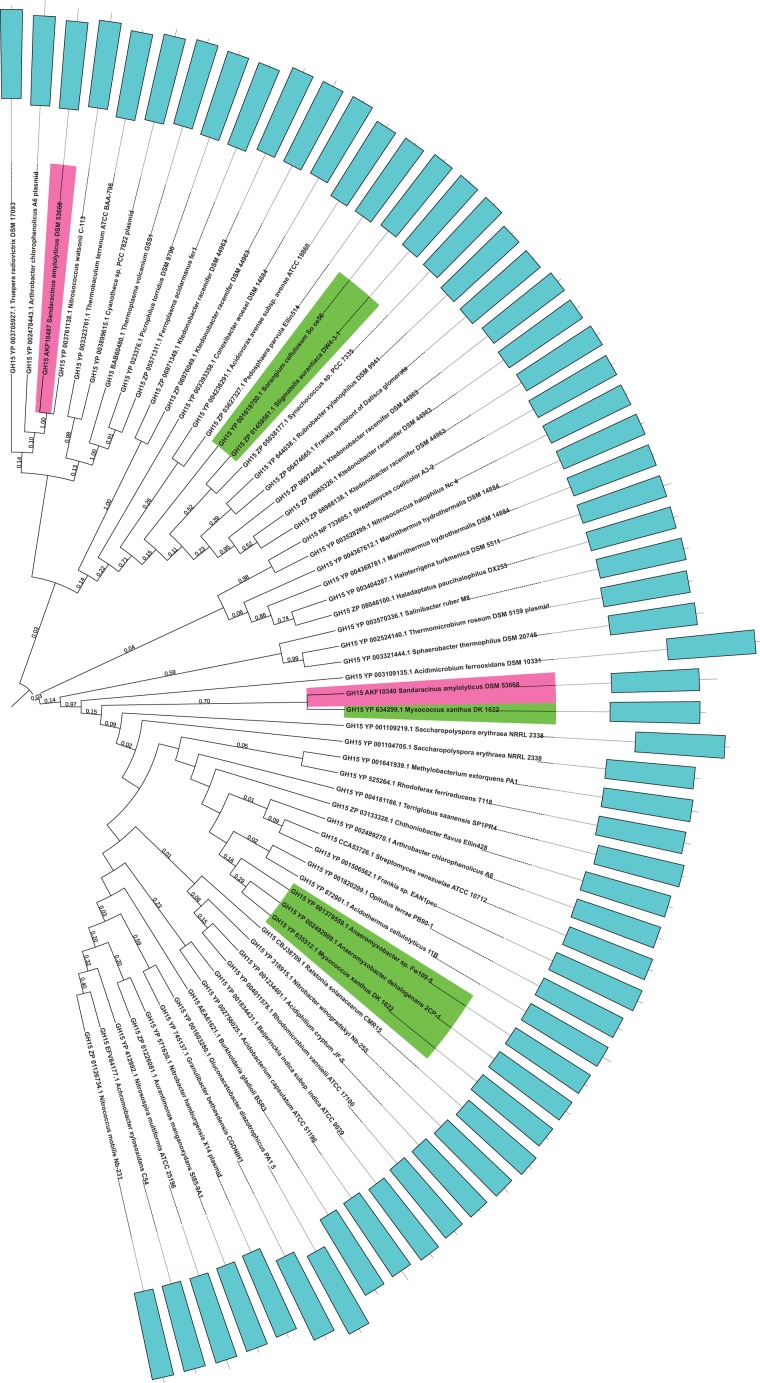
Phylogeny of *Sandaracinus amylolyticus* GH15 γ-amylases*. Sandaracinus amylolyticus* γ-amylases are colored dark pink and myxobacterial homologs are colored fluorescent green. Bootstrap values corresponding to the tree nodes are provided.

Similarly, the phylogenetic inference was drawn from two representative domains of γ-amylases using the same methodology as followed for α-amylases (fig. 4). We found that one of the γ-amylases AKF10340 lies closer to *M. xanthus* amylase whereas the other AKF10487 shares its sister branch with *Nitrosococcus watsonii*, a Gammaproteobacteria.

This suggests that while some of the α- and γ-amylases are present in many myxobacterial species, a majority of the α- and γ-amylases are present only in *S. amylolyticus*, and not in other myxobacterial genomes, possibly due to the result of horizontal transfer events from diverse bacterial phylum such as Deinococcus-Thermus, Cyanobacteria, Alphaproteobacteria, Gammaproteobacteria, and Acidobacteria.

### Agar Degrading Enzymes

Some bacteria are known to degrade agar and use it as a carbon source and possess agarase enzymes that help in hydrolyzing agar in oligosaccharides (Chi et al. 2012). Depending on the site of action, they can be of two types; α-agarase (agarose 3-glycanohydrolase; 3.2.1.158; GH96) and β-agarase (agarose 4-glycanohydrolase; 3.2.1.81: GH16, GH50, GH86, GH118) (table 1). Both families of agarases were absent in *S. amylolyticus* genome which could be related to its agar nondegrading property (Mohr et al. 2012) as supported by colony morphology where agar was not penetrated or liquefied by the bacteria (supplementary fig. S5, Supplementary Material online**)**.

**Table 1:** **Cellulose, Starch, Chitin, and Agar Degrading Enzymes and Their Representative CAZY Domains Distribution**

Substrate	Enzyme Category	Enzyme Name	EC Number	CAZY Families (protein repertoire in brackets)
Starch	Amylase	α-Amylase	3.2.1.1	**GH13 (15)**, **GH57 (1)**, GH119, GH126
	Amylase	β-Amylase	3.2.1.2	GH14
	Amylase	γ-Amylase	3.2.1.3	**GH15 (2)**, GH97
Chitin	Chitinase	Chitinase	3.2.1.14	GH18, **GH19 (2)**, **GH23 (5)**, GH48
Agar	α-Agarase	Agarose 3-glycanohydrolase	3.2.1.158	GH96
	β-Agarase	Agarose 4-glycanohydrolase	3.2.1.81	GH16, GH50, GH86, GH118
Cellulose	Cellulase	β-Glucosidase	3.2.1.21	**GH1 (3)**, **GH3 (2)**, **GH5 (2)**, GH9, GH30, GH116
	Cellulase	Cellobiohydrolase or Exoglucanase	3.2.1.176	GH48, GH6, GH7, GH9
	Cellulase	Endoglucanase	3.2.1.4	**GH5 (2)**, GH6, GH7, GH8, GH9, GH12, GH44, GH45, GH48, GH51, **GH74 (3)**, GH124

Note.—EC number, enzyme category and CAZY families for proteins involved in starch, chitin, agar, and cellulose degradation are shown. The number of *Sandaracinus amylolyticus* proteins identified having CAZY domains are represented in bold fonts in respective families.

### Cellulose Degrading Enzymes

We identified only GH5 and GH74 in *S. amylolyticus* proteome in two and three proteins, respectively. GH5 domain was present as a single unit in AKF04963 and AKF09194 whereas GH74 was identified in AKF04859, AKF06963, and AKF09775 associated with a GH74 domain. CAZY domains for cellobiohydrolase/exoglucanases (represented by GH6, GH7, GH9, and GH48) were not present in *S. amylolyticus*. Cellobiohydrolase/exoglucanases plays a significant role in cellulose degradation as they cut two to four sugar subunits from the exposed ends formed by the endoglucanases, which are used as initial substrates for β-glucosidase (Mba Medie et al. 2012). The absence of cellobiohydrolase or exoglucanases could be a possible reason for the lack of cellulose degradation in *S. amylolyticus*. Out of β-glucosidase representative CAZY families GH1, GH3, GH5, GH9, GH30, and GH116, only GH1, GH3, and GH5 families were found in three, two and two proteins in *S. amylolyticus*, respectively. AKF03843, AKF06028, and AKF10711 have a GH1 domain whereas AKF09078, and AKF10875 have only the GH3 domain.

### Chitin Degrading Enzymes

Chitinases (EC:3.2.1.14) are involved in degradation of chitin, a component of the cell wall of fungi and exoskeleton of arthropods. GH18, GH19, GH23, and GH48 families have been reported in the CAZY database as chitinases that hydrolyse β-1,4 linkages between N-acetylglucosamine units. GH18 is a chitinase family with a characteristic “[LIVMFY][DN]G[LIVMF][DN][LIVMF][DN]E” motif and is expressed in archaea, prokaryotes, and eukaryotes (Funkhouser and Aronson 2007). GH18 chitinases were not found in *S. amylolyticus* genome while members of GH19 (AKF03006 and AKF10423) and GH23 (AKF04575, AKF05533, AKF06346, AKF08835, and AKF11377) could be identified. GH19 family chitinases are mostly reported to be present in plants with only a few members in prokaryotes (Kasprzewska 2003; Funkhouser and Aronson 2007). Among the two identified GH19 homologs, we found that AKF10423 is approximately 65% identical (∼68% query coverage) with cyanobacteria such as *Nodosilinea nodulosa* and *Limnoraphis robusta* along with another myxobacterium *Chondromyces crocatus*. Besides these homologs, we also found that AKF10423 shows maximum similarity with eukaryotic GH19 chitinases counterparts as present in *Acacia mangium*, *Festuca arundinacea*, *Triticum aestivum*, and *Leucaena leucocephala* (55–59% identity with 70% query coverage). We identified that another chitinase, AKF06346, has GH23 domain along with three copies of CBM50, which is generally present in the enzymes cleaving either chitin or peptidoglycan.

## Conclusion

The complete 10.33 Mb circular genome of *S. amylolyticus* type strain DSM 53668^T^ was determined. CAZy analysis revealed the presence of 72 GT domains, 72 CE domains, 58 GH domains, 19 AA domains, 4 PL domains, and 26 CBM domains. Sixteen *S. amylolyticus* proteins were identified of which 15 are α-amylases of class GH13 and 1 GH57. Two γ-amylases of GH15 family were also identified. Phylogeny analysis revealed that ten GH13 α-amylases and one GH15 γ-amylase have been aquired via horizontal gene transfer from other bacterial phyla such as Deinococcus-Thermus, Cyanobacteria, Alphaproteobacteria, Gammaproteobacteria, and Acidobacteria.

## Supplementary Material

Supplementary figures S1–S5 and tables S1 and S2 are available at *Genome Biology and Evolution* online (http://www.gbe.oxfordjournals.org/).

Supplementary Data
