# Supplementary material for: Complete Genome of the Starch-Degrading Myxobacteria Sandaracinus amylolyticus DSM 53668T
Source: Genome Biol Evol. 2016 Jun 29;8(8):2520–9. doi: 10.1093/gbe/evw151 (PMC5010890; doi:10.1093/gbe/evw151)
Supplement: Supplementary Data [file supp_8_8_2520__index.html]

Complete Genome of the Starch-Degrading Myxobacteria Sandaracinus amylolyticus DSM 53668T — Supplementary Data 

# Complete Genome of the Starch-Degrading Myxobacteria *Sandaracinus amylolyticus* DSM 53668T

## Supplementary Data

files

- Supplementary Data - zip file
