# Supplementary material for: Complete Genome of the Starch-Degrading Myxobacteria Sandaracinus amylolyticus DSM 53668T
Source: Genome Biol Evol. 2016 Jun 29;8(8):2520–9. doi: 10.1093/gbe/evw151 (PMC5010890; doi:10.1093/gbe/evw151)
Supplement: Supplementary Data [file supp_evw151_suppl_data.zip › Figure-S2.pdf]

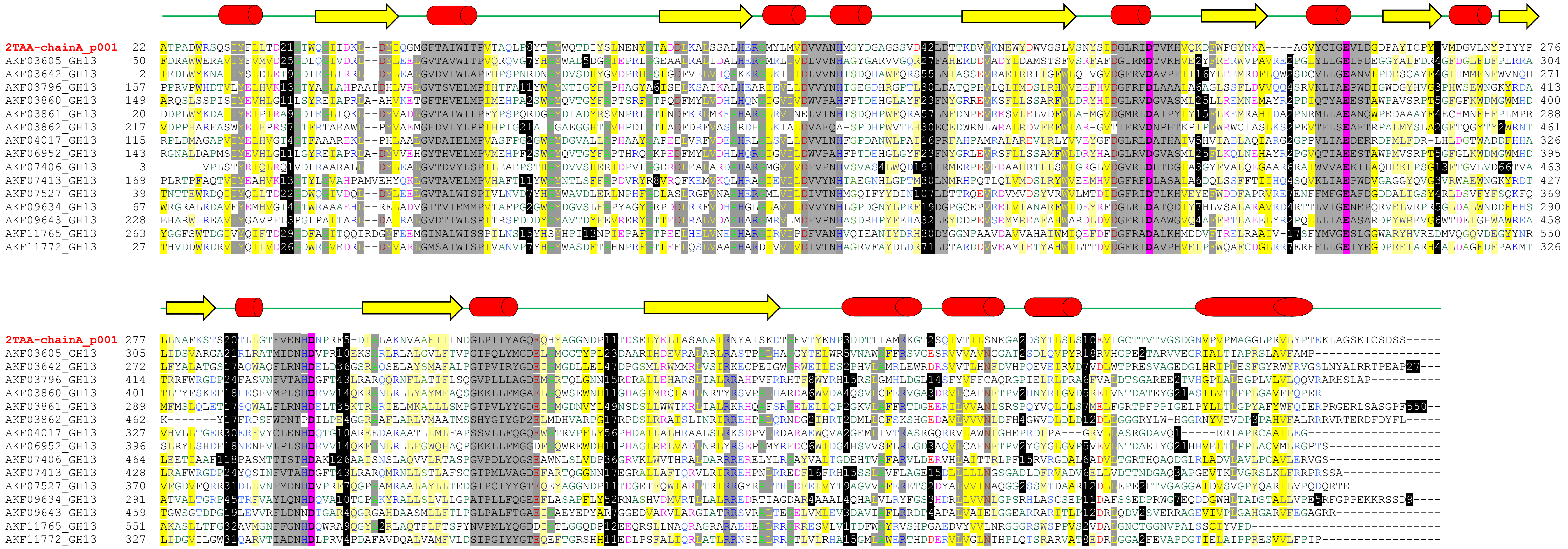

2TAA-chainA_p001 22 276
AKF03605_GH13 50 304
AKF03642_GH13 2 271
AKF03796_GH13 157 413
AKF03860_GH13 149 400
AKF03861_GH13 20 288
AKF03862_GH13 217 461
AKF04017_GH13 115 326
AKF06952_GH13 143 395
AKF07406_GH13 3 463
AKF07413_GH13 169 427
AKF07527_GH13 39 369
AKF09634_GH13 67 290
AKF09643_GH13 228 458
AKF11765_GH13 263 550
AKF11772_GH13 27 326
2TAA-chainA_p001 277
AKF03605_GH13 305
AKF03642_GH13 272
AKF03796_GH13 414
AKF03860_GH13 401
AKF03861_GH13 289
AKF03862_GH13 462
AKF04017_GH13 327
AKF06952_GH13 396
AKF07406_GH13 464
AKF07413_GH13 428
AKF07527_GH13 370
AKF09634_GH13 291
AKF09643_GH13 459
AKF11765_GH13 551
AKF11772_GH13 327
