# Supplementary material for: Complete Genome of the Starch-Degrading Myxobacteria Sandaracinus amylolyticus DSM 53668T
Source: Genome Biol Evol. 2016 Jun 29;8(8):2520–9. doi: 10.1093/gbe/evw151 (PMC5010890; doi:10.1093/gbe/evw151)
Supplement: Supplementary Data [file supp_evw151_suppl_data.zip › Figure-S3.pdf]

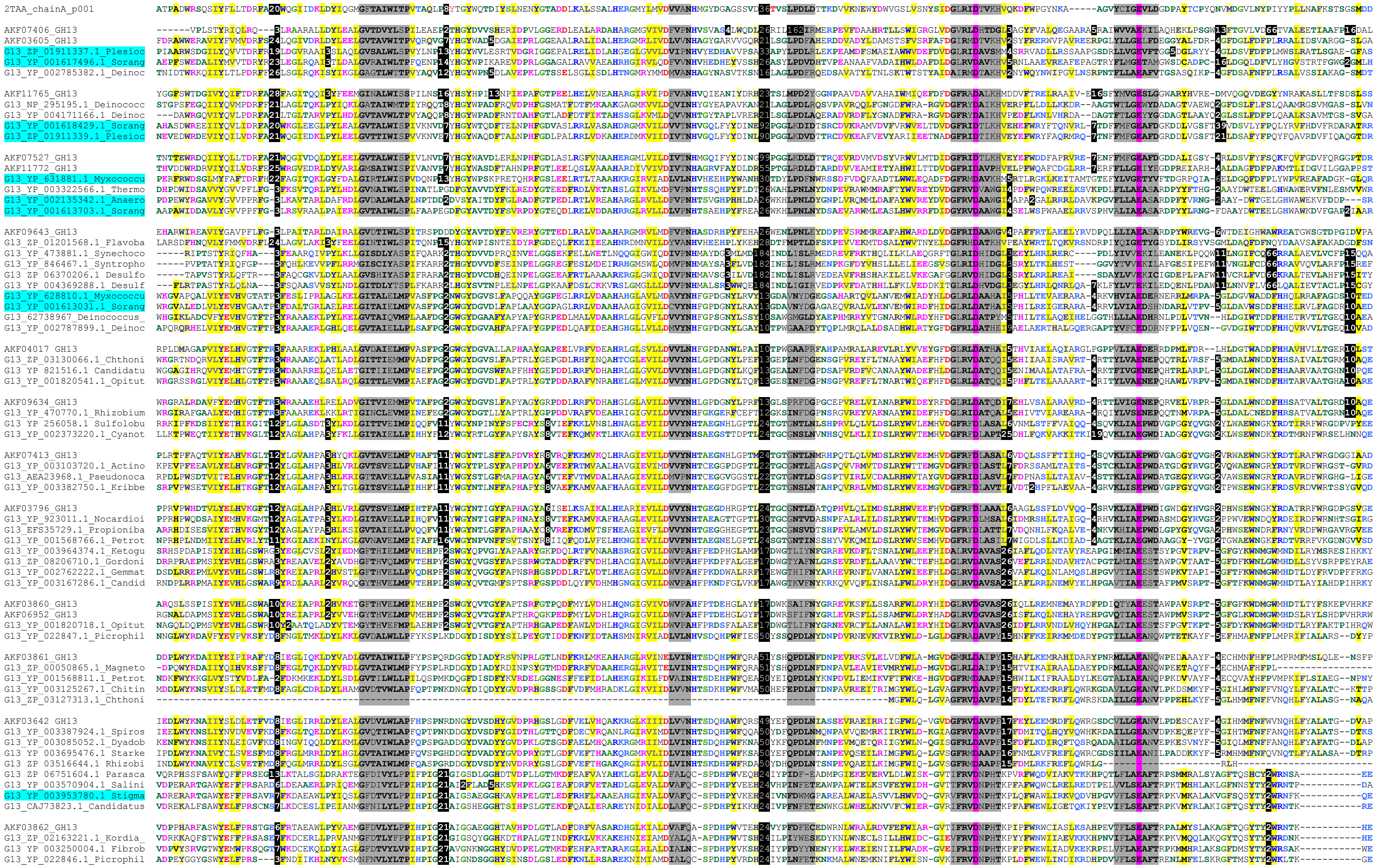
2TAA_chainA_p001
AKF07406_GH13
AKF03605_GH13
G13_ZP_01911337.1_Plesioc
G13_YP_001617496.1_Sorang
G13_YP_002785382.1_Deinoc
AKF11765_GH13
G13_NP_295195.1_Deinococc
G13_YP_004171166.1_Deinoc
G13_YP_001618429.1_Sorang
G13_ZP_01911339.1_Plesioc
AKF07527_GH13
AKF11772_GH13
G13_YP_631881.1_Myxococcu
G13_YP_003322566.1_Thermo
G13_ZP_02135342.1_Anaero
G13_YP_001613703.1_Sorang
AKF09643_GH13
G13_ZP_01201568.1_Flavoba
G13_YP_473881.1_Synechoco
G13_YP_846467.1_Syntropho
G13_ZP_06370206.1_Desulfo
G13_YP_004369288.1_Desulf
G13_YP_628810.1_Myxococcu
G13_YP_001613031.1_Sorang
G13_62738967_Deinococcus_
G13_YP_002787899.1_Deinoc
AKF04017_GH13
G13_ZP_03130066.1_Chthoni
G13_YP_821516.1_Candidatu
G13_YP_001820541.1_Opitut
AKF09634_GH13
G13_YP_470770.1_Rhizobium
G13_YP_256058.1_Sulfolobu
G13_YP_002373220.1_Cyanot
AKF07413_GH13
G13_YP_003103720.1_Actino
G13_AEA23968.1_Pseudonoca
G13_YP_003382750.1_Kribbe
AKF03796_GH13
G13_YP_923011.1_Nocardioi
G13_EFS35729.1_Propioniba
G13_YP_001568766.1_Petrot
G13_YP_003964374.1_Ketogu
G13_ZP_08206710.1_Gordoni
G13_YP_002762222.1_Gemmat
G13_YP_003167286.1_Candid
AKF03860_GH13
AKF06952_GH13
G13_YP_001820718.1_Opitut
G13_YP_022847.1_Picrophil
AKF03861_GH13
G13_ZP_00050865.1_Magneto
G13_YP_001568811.1_Petrot
G13_ZP_03125267.1_Chitin
G13_ZP_03127313.1_Chthoni
AKF03642_GH13
G13_YP_003387924.1_Spiros
G13_YP_003085052.1_Dyadob
G13_YP_003695476.1_Starke
G13_ZP_03516644.1_Rhizobi
G13_ZP_06751604.1_Parasca
G13_YP_003570904.1_Salini
G13_YP_003953780.1_Stigma
G13_CAJ73823.1_Candidatus
AKF03862_GH13
G13_ZP_02163221.1_Kordia_
G13_YP_003250004.1_Fibrob
G13_YP_022846.1_Picrophil

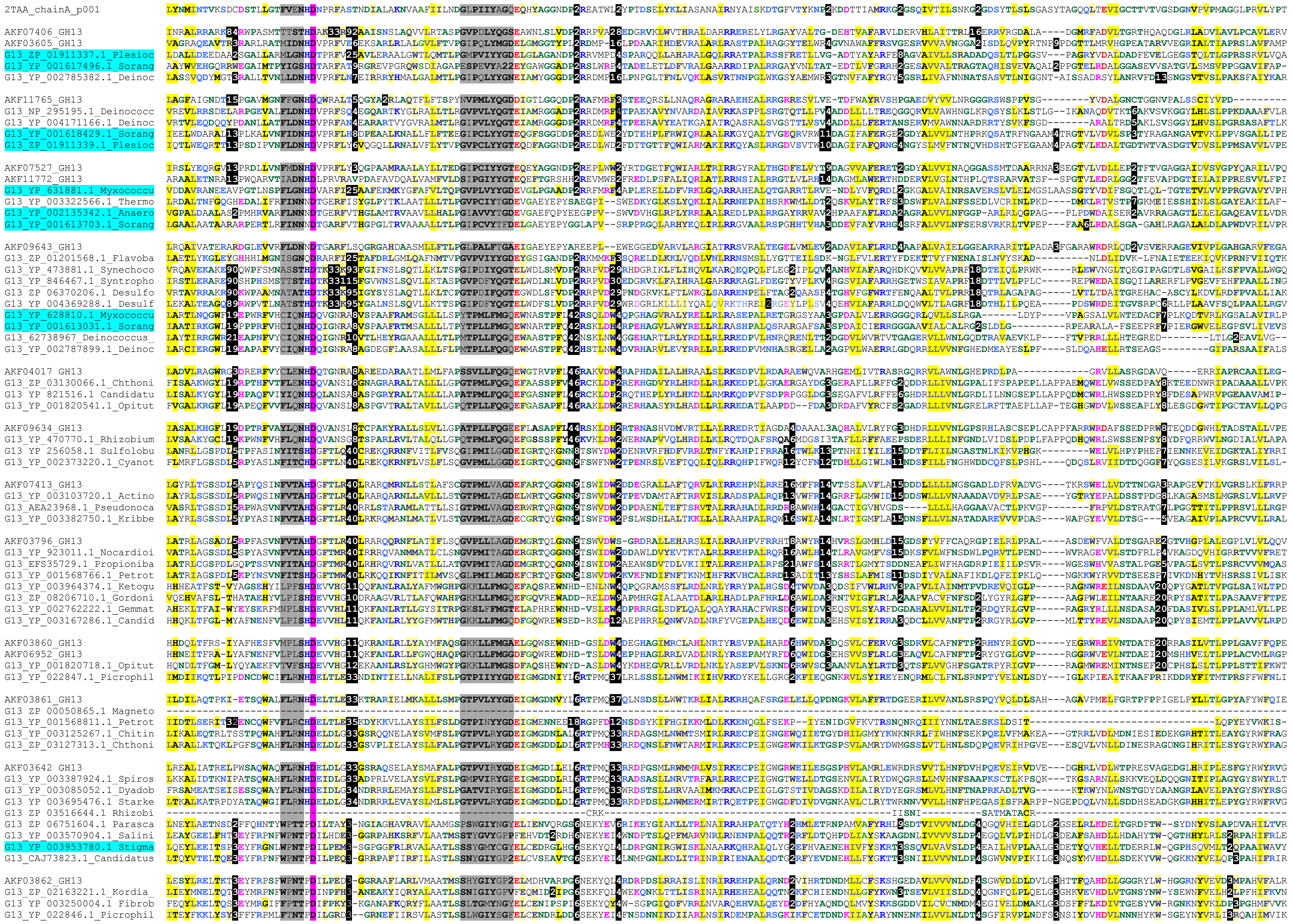

2TAA_chainA_p001
AKF07406_GH13
AKF03605_GH13
G13_ZP_01911337.1_Plesioc
G13_YP_001617496.1_Sorang
G13_YP_002785382.1_Deinoc
AKF11765_GH13
G13_NP_295195.1_Deinococc
G13_YP_004171166.1_Deinoc
G13_YP_001618429.1_Sorang
G13_ZP_01911339.1_Plesioc
AKF07527_GH13
AKF11772_GH13
G13_YP_631881.1_Myxococcu
G13_YP_003322566.1_Thermo
G13_YP_002135342.1_Anaero
G13_YP_001613703.1_Sorang
AKF09643_GH13
G13_ZP_01201568.1_Flavoba
G13_YP_473881.1_Synechoco
G13_YP_846467.1_Syntropho
G13_ZP_06370206.1_Desulfo
G13_YP_004369288.1_Desulf
G13_YP_628810.1_Myxococcu
G13_YP_001613031.1_Sorang
G13_62738967_Deinococcus_
G13_YP_002787899.1_Deinoc
AKF04017_GH13
G13_ZP_03130066.1_Chthoni
G13_YP_821516.1_Candidatu
G13_YP_001820541.1_Opitut
AKF09634_GH13
G13_YP_470770.1_Rhizobium
G13_YP_256058.1_Sulfolobu
G13_YP_002373220.1_Cyanot
AKF07413_GH13
G13_YP_003103720.1_Actino
G13_AEA23968.1_Pseudonoca
G13_YP_003382750.1_Kribbe
AKF03796_GH13
G13_YP_923011.1_Nocardioi
G13_EFS35729.1_Propioniba
G13_YP_001568766.1_Petrot
G13_YP_003964374.1_Ketogu
G13_ZP_08206710.1_Gordoni
G13_YP_002762222.1_Gemmat
G13_YP_003167286.1_Candid
AKF03860_GH13
AKF06952_GH13
G13_YP_001820718.1_Opitut
G13_YP_022847.1_Picrophil
AKF03861_GH13
G13_ZP_00050865.1_Magneto
G13_YP_001568811.1_Petrot
G13_YP_003125267.1_Chitin
G13_ZP_03127313.1_Chthoni
AKF03642_GH13
G13_YP_003387924.1_Spiros
G13_YP_003085052.1_Dyadob
G13_YP_003695476.1_Starke
G13_ZP_03516644.1_Rhizobi
G13_ZP_06751604.1_Parasca
G13_YP_003570904.1_Salini
G13_YP_003953780.1_Stigma
G13_CAJ73823.1_Candidatus
AKF03862_GH13
G13_ZP_02163221.1_Kordia_
G13_YP_003250004.1_Fibrob
G13_YP_022846.1_Picrophil
