# Supplementary material for: Complete Genome of the Starch-Degrading Myxobacteria Sandaracinus amylolyticus DSM 53668T
Source: Genome Biol Evol. 2016 Jun 29;8(8):2520–9. doi: 10.1093/gbe/evw151 (PMC5010890; doi:10.1093/gbe/evw151)
Supplement: Supplementary Data [file supp_evw151_suppl_data.zip › Figure-S4.pdf]

```
>1K1W_chainA_p001              INFIFGIHNH--QPLGNFGWVFEEAYNRSYRPFMEILEEFP3VNVHFSGPLLEWIEENKPDYLDLLRSLIKRGQLEIVVAGFYEPVLAAIPKEDRLVQIEMLKD-YARKLGYDAKGVWLTERVWQPELVKSLREAGIEYVVVDDYHFMSAGLSKEELFWPYYTE-DGGEVITVFPIDEKLRYLIPFRPVKKTIEYLESLSKVAVFHDDGEKFGVWP--G
>AKF06951__GH57                M3KYICIHGHFYQPPRENPWL17RVFAECYGPN14IVNNYASISFNFGPTLLSWMEQAQPEVYDAVLAAD7GGHGSAMAQVYGHVILPLANERDKRTQVIWGVRDFERRFGRAPRGMWLSETAADVASLEALAEQGIRFTVLAPNQ13DVSGSRVDTRRAYEVALPSGRSIAVFFYDGPTSRAVAFE5GKTFARRLASV7QLVHIATDGESYGHHHRFG
>G57_YP_001378953.1_Anaero     M2RLLCLHGHFYQPPRENPWI17RIAAECYAPN14IVNNYVHLSFNFGPTLLAWLERERPDVHARVLEAD6RGHGNALAQGFNHAILPLASARDRLTQIRWGLADFRRRFGRAPEGFWLPETAADTPTLEVLAQEGIRYTVLSPYQ13DATGARFDPTRPYRVRA-GDRELVVFFYDGHIARDLAFG5PQALLRRLEGG8EILTIALDGETLGHHKKGG
>G57_YP_002463416.1_Chloro     M2RLICIHGHFYQPPRENPWL17RITAECYEQN14IVNNYSRISFNFGPTLLTWLAAHAPQVYQAILQAD6FGAGSAMAQCYNHIIMPLASRRDKVTQVIWGIRDFVHRFGREPEGMWLPETAVDLETLDIMAEHGIKFTILAPTQ13DVSGGRIDPTQPYLVKLASGRAITVFFYDGPVSRAVAFE5GVGFANRLASI8QLAHIATDGETYGHHHRHG
>G57_YP_002951636.1_Desulf     MDRALCIHGHFYQPPREDPWL17RICRESYAPM14LFNCYSHMSFNAGPTLMSWMARSAPDTYARILEAD6LGHGNALAQIYHHVIMPLATERDKRLEVAWAVDDFKARFGRDPEGMWLSETAVDTPTLEALAQAGIRFTILAPSQ12GVDAGSLDIRRPYAVRLPSGREIAVFFYHGPLSQAVAFE5GEHFFRRLSES3GLLSLATDGETYGHHFKFG
>G57_YP_003806467.1_Desulf     MAKHVCVHGHFYQPFRENPWL17RVAAECYAPN14IVNNYQFMSFNFGATLMGWLRAQAPAVHEALIEAD6WGHGGAMAQVYHHLIMPLCNDRDKQTQILWGLADFAHRFGRPAAGMWLAETAVDTPTLELMAQAGVKFTVLAPRQ13EIGADGLNTRRPYIVRLRGGRSIVVFFYDGPVSQAVAFE5GARFAQRIMGA8QLVSLATDGESYGHHHRFG
>G57_YP_004369289.1_Desulf     MTKYICIHGHFYQPPRENPWL17RITAECYAPN14IPNNYAKISFNFGPTLLAWLEQKTPAVYESILLAD8SGHGSALAQCYNHLIMPLANTRDKYTQVVWGIRDFENRFQRQPEGMWLPETAVDLETLDILAAAGIKFTILAPRQ13EVSGERIDPTRAYLQRLPSGREIALFFYDGPISRAVAFE5GEYLAERLLSA8QLIHIATDGETFGHHHRHG
>G57_ZP_06391561.1_Dethios     MSRYVCIHGHFYQPPRENPWT17RIADQCYGPC14TRNCYGSMSFNVGPTLLGWLEDRRPWIYRSILEAD8EGHGPAITQVYGHAILPLASERDRRTQVMWGISDFVRRFRRRPEGMWLPEAAVDTESLEVLAGEGIKFALLAPHQ-3SASDGPIDISKPYICMLPSGAKISLFFYDGELAREMAFG5GRRLARRLSDR7ALEHVAVDGETFGHHHRFG

>1K1W_chainA_p001              TYWLREFFDAITEKINLMTYSEYLSKFTPRGLVYL6EMSEWSLPAKQAKLFVEFVEQLKEEGK7VRGGIWKNFFFKYPESNFMHKRMLMVSKAVRDNPEARKYILKAQCNDAYWHGVFGGIYLPHLRRTVWENIIKAQRYLK22FIATIKPHYGGSIFELSSKRKAVNYNDVLPR23RELAYDWQLRA30PYEYEMIENGVKLWREGGVYAE2IPARVE
>AKF06951__GH57                EMALAYALQTIER3VRLTTYEEFLDRHPPEWQAEIVPNTSWSCAH39VEVYEREALGLLRDPWAARDDYVDVVLDRGDDALRRFFGRHGAP13ALELLEMQRHAMLMYTSCAWF5GLETVQCILYAARAVQLATEIAGDKFEAGFLARLA9GDGERIYVERVRPAMVDLRKVGAHFAASSLFEEYRARERVFGFDVDVTDREEARAGQARMSIGRMEVR
>G57_YP_001378953.1_Anaero     DEVLAAALRLLAR3VELVNLGQALDRIAPEWEAEIAEGSSWSCAH39ADLYQREAASLLADPWGARDRFVEVVLDPDRRESEEFLRREAGR8KALRLLELQRQSLLMFTSCGWF5GIETVQVMKYAARAIQLARDAAGVDLEPGFVAALE9GDGRRVYEALVRPSVVSLEGVGAHLAIASSVRETPPEGRLFCFRYRLEAGRSAQSGPATLATGRLHLE
>G57_YP_002463416.1_Chloro     DMALAYALHYIEE3AKLTNYAAYLQRYRPTHEVQIIERTSWSCAH39APRFEGYARRLLHDPWAARDDYISVILDRSPENVAAFIGRHSRG8AILKLMELQRHLMLMYTSCGWF5GIETIQVMMYAGRAIQLAHELFGEEIEGEFLNRLA9GNGRDLYERHVRPAMVDLRKVGAHYAMTALFNGVGEHEQIYAYTVEREDYHLLLAGKSRLALGRIRII
>G57_YP_002951636.1_Desulf     EMALAYALDKAKS4LTLTNFAAYLAANPPKAYAKIREASAWSCAH39DSHYDAKAGALFKAPEAALDAYGQVLAGTQS--KEGFERLHMAP8AAWKLLSMQAWGLASFASCAWF5RLEPVNAMTFALRAVELARATGMADPEPEILTILA9GSGADIWETMARPRRETPKTLITQALVTLALEN2PLGGEEAAATWPGVAV5NAPAGAPRPGAAAIVYR
>G57_YP_003806467.1_Desulf     EMALAFALQRIDH3VKLTNFAAYLAANPPTWEARIVEASSWSCAH39ADLFEQKADGLLHDPWAARDDYVRLLLDNSAAGRKRFLENHAKI8TVWQLLEMQRWAMAMFTSCGWF5GLEPVQNLRMAARAIQLARELGAAGLERSLVATLS9GSGADIWRARVAPELVGLERVAAHAAISGVMEEEPPPPRLYCYQLADLGHRHIDHLGASLSWGALRAR
>G57_YP_004369289.1_Desulf     EMALSYALEYIEA3VRLINYGEYLEKYPPAMEVEIFENSSWSCIH39INLFEEKASVLLKDPWAARNDYLEVIFSRSPESVQSLLEHHALK8EVLRLLEIQRCAQLMYTSCGWF5GIETVQVIQYAGRAIQLAQELSADDLEEGFLTRLE9ADGRCIYQKFVKPAAVGLLDVGAHYAISSLFEDYGEQDRIFYYDIHRRDYRRAEAGKATLVIGRIEVS
>G57_ZP_06391561.1_Dethios     EMALAACLDELDR3LDLTVYGRFLEICPPESEVEIVERSSWSCVH39DELFQEKGASLFSDPWAVRDRSERLYSKLSPLDRIAFLVDEAGR8LGLSLLEMERCSMLMFSSCGWF5RIEAIQVLRYASKVLALTERLGKSGLRKPFLEAALE9GNGSRIFSFFVEPASMDLPRVGAHVALYRLFDLDPGETEFPLISVKDGTVCRCSSDGEERFFGQLTVR

>1K1W_chainA_p001              KKIELTEDGFIAKYRVLLEK23FEAKEFEVNDPYGIGKV4DKAAKVWKFPIKTLSQSEAGWDFIQQGVSYTMLF--------------------PIEKELEFTVRFREL
>AKF06951__GH57                SRVTQAS14LSGGVRAWQGDAAHASLHNALRAAFGRADI7ERSFPNATYTLRTLF19ERDYEQIYA24EYTLTARLRRELERG2VDLEGARALLQEARDAGIVVQRDEL
>G57_YP_001378953.1_Anaero     SMITRES14FRAGLLPYPGPEAHAEIERVLFAKLERISF7DRAFPGRDYALRDLF19EDDYLRIYD24DVTLTRRILEITGRA8EGERELLATVELARRLGAHFDVVAV
>G57_YP_002463416.1_Chloro     SNITGES14ISGGVREYQNEQIYQQLIEELSELFLRADI7DRNFGQEQYSLKLLF19EAAYRQIYE24EFAINTELRRLFETE2LDFDRINSLLREAQRSGVTLDAEGL
>G57_YP_002951636.1_Desulf     HETAADR28EGF-----------------------------------AFEAVGLP19HAENLAAAA20PLVLHLW5LEVFEPP3KKRELLHLFLKHTGDG------SPV
>G57_YP_003806467.1_Desulf     HVRIGRD14FAAWVRPRPTDWDIDEIGAAAEDLLRSLEL7ARLVGGQRFDLSDLF19FEVAKSIYD24EAMIGEELVQGMDLP3DLAGHLGALARQAKALSLKPNGGRL
>G57_YP_004369289.1_Desulf     SRITLHS14ICGGVRFFQGQKAYSLMTREMTKTFAGADF7DRHFGASTYSLRHLF19QGTYQQVYK24ELVINWNLRQALEAE2PDMEVIGSLLNDARVFDVQIDAATL
>G57_ZP_06391561.1_Dethios     SELTGRS14ILCGALPITEGADPEVLMERVRFASNC---6GSIFGHRLYSLRHLF19ALAVKDVVL19KIFVDYRLNRELSMK2PSYGDLLDLLGRSRRWGVQLDRERL
```
