# Supplementary figures and images for: Complete Genome of the Starch-Degrading Myxobacteria Sandaracinus amylolyticus DSM 53668T
Source: Genome Biol Evol. 2016 Jun 29;8(8):2520–9. doi: 10.1093/gbe/evw151 (PMC5010890; doi:10.1093/gbe/evw151)

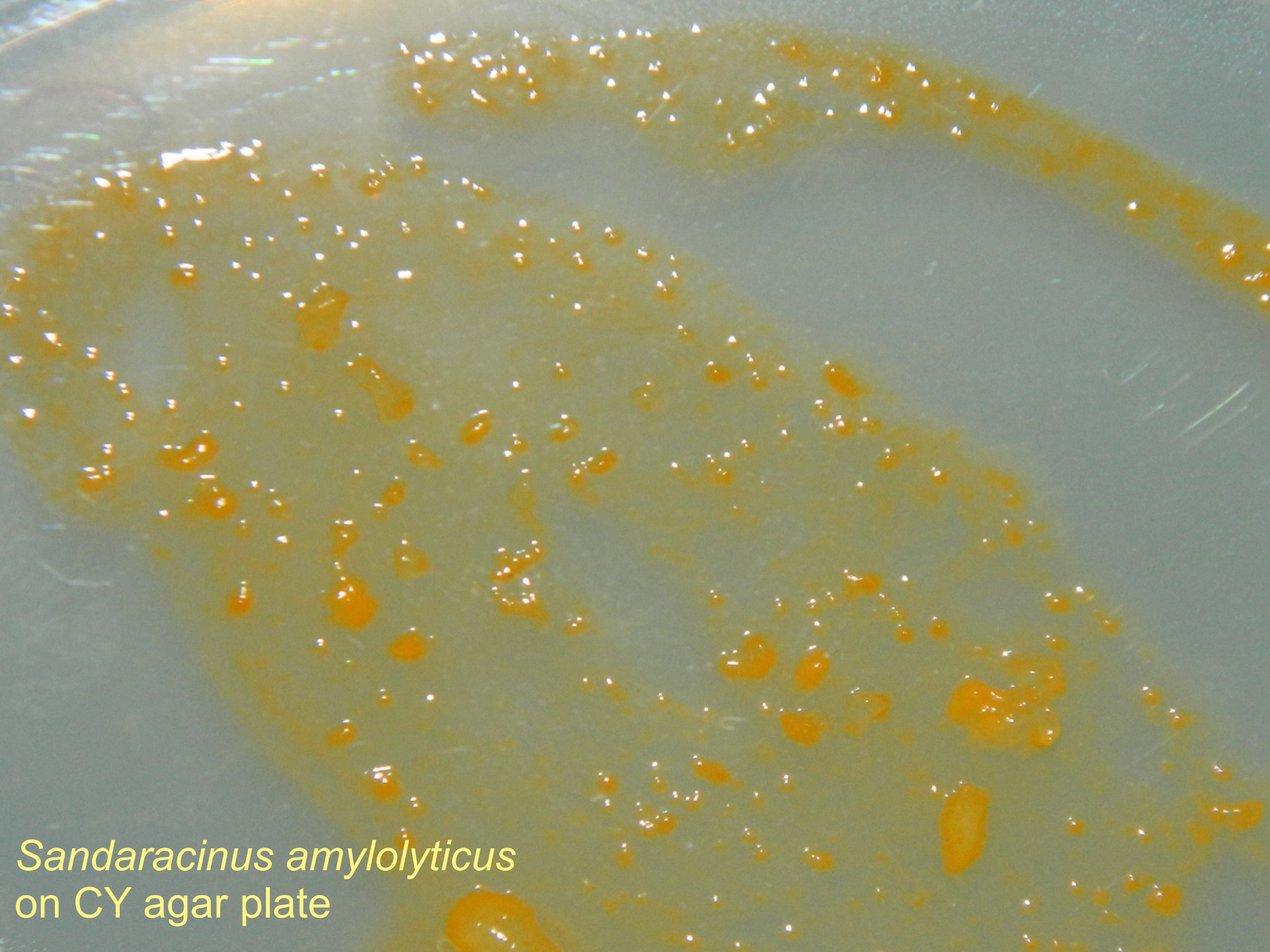

*Sandaracinus amycolyticus*  
on CY agar plate

Supplement: Supplementary Data [file supp_evw151_suppl_data.zip › Figure-S5.pdf]

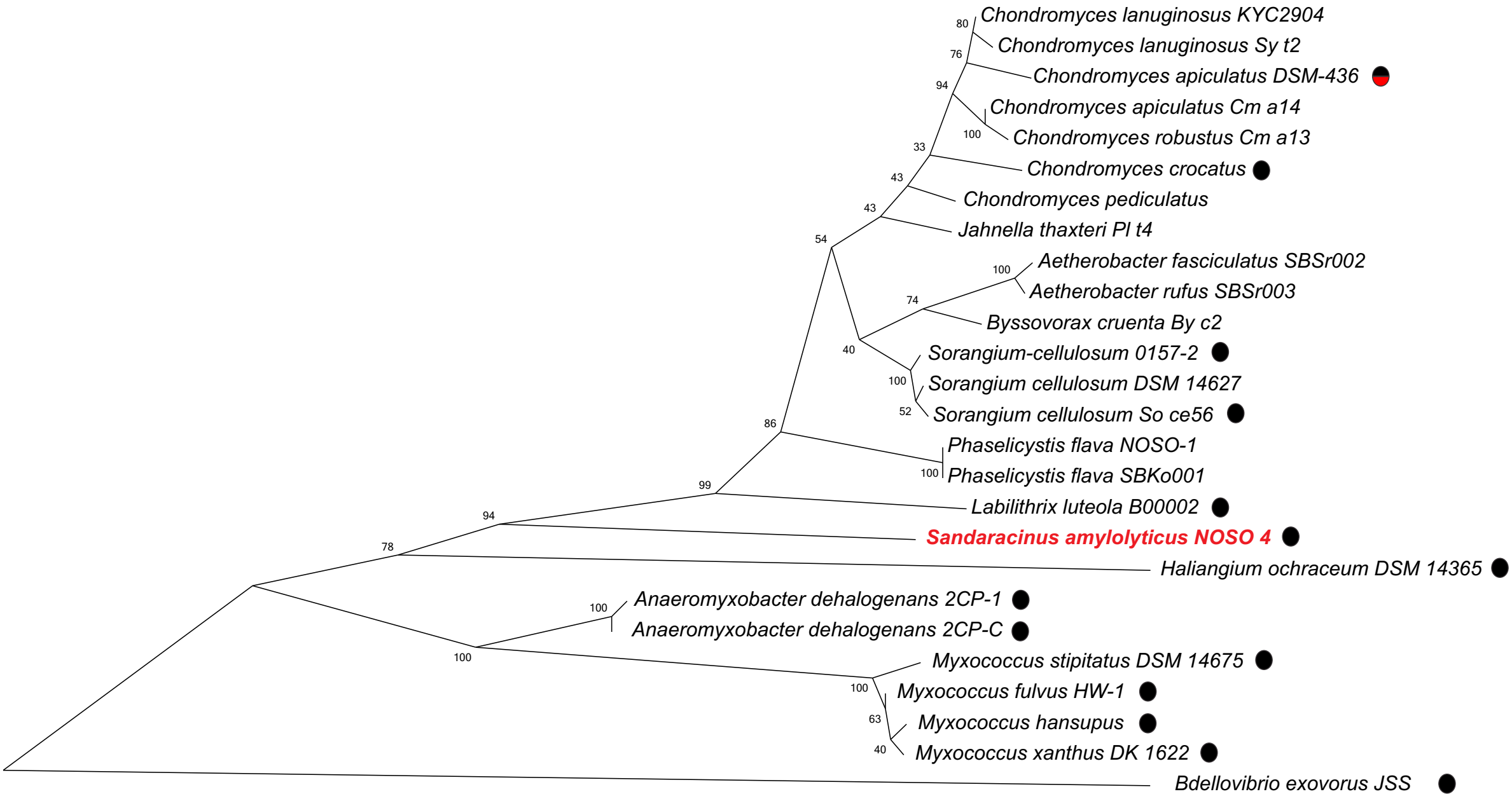

0.05

Supplement: Supplementary Data [file supp_evw151_suppl_data.zip › Figure-S1.pdf]
